# Spatial Access Matters: An Analysis of Policy Change and Its Effects on Avoidable Infant Mortality in Portugal

**DOI:** 10.3390/ijerph18031242

**Published:** 2021-01-30

**Authors:** Morgan Weiland, Paula Santana, Claudia Costa, Julia Doetsch, Eva Pilot

**Affiliations:** 1 Department of Health, Ethics and Society, Faculty of Health, Medicine and Life Sciences (FHML), Care and Public Health Research Institute (CAPHRI), Maastricht University, 6220 MD Maastricht, The Netherlands; morganweiland@btinternet.com (M.W.); julia.nadine.doetsch@gmail.com (J.D.); 2 Centre of Studies in Geography and Spatial Planning (CEGOT), University of Coimbra, 3004-531 Coimbra, Portugal; paulasantana@uc.pt (P.S.); claudiampcosta@uc.pt (C.C.); 3 EPIUnit–Instituto de Saúde Pública, Universidade do Porto, Rua das Taipas, n° 135, 4050-600 Porto, Portugal

**Keywords:** avoidable infant mortality, spatial inequalities, healthcare access, maternity unit, healthcare service, Portugal

## Abstract

In 2006, a policy reform restructured the maternal and perinatal healthcare system, including closing smaller maternity units, to further improve care in Portugal. This study aimed to investigate the effects of the 2006 National Program of Maternal and Neonatal Health policy on spatial inequalities in access to care and consequently avoidable infant mortality. A thematic analysis of qualitative data including interviews and surveys and a quantitative spatial analysis using Geographic Information Systems was applied. Spatial inequalities were found which may lead to avoidable infant mortality. Inequalities exist in freedom of choice and autonomy in care, within a medicalized system. Changes in approach to and organization of care would further enhance equitable spatial access to care in maternal health and reduce avoidable infant mortality.

## 1. Introduction

By promoting universal and general access and public insurance, the Portuguese National Health Service (NHS) aims to ensure equity, efficiency, and quality of health care in all services [1]. However, the current system is not achieving these objectives, particularly with respect to financial and geographic equity of access [2,3]. Progress is hampered by excessive concentration of public and private resources in urban areas, resource allocation based on outdated indicators and predominance of the curative model [4].

The NHS is a universal tax-financed system which coexists with special private insurance schemes for certain professions or companies and private voluntary health insurance [5]. Planning and regulation of the NHS is undertaken centrally by the Ministry of Health while management occurs regionally through regional health administrations [6]. Private prenatal care is accessed frequently by women in Portugal and is covered by either out-of-pocket payments or employer-sponsored health insurance schemes [7].

Maternal and perinatal care plays a significant role in reducing infant mortality. Infant mortality rate (IMR) is defined as the ratio of deaths in the first year of life to the number of live births in a given year and is expressed per 1000 live births [8]. The reduction in IMR on a global scale has been the most important factor in improving life expectancy over recent decades, from 64.7 in 1990 to 28.9 in 2018 [9,10]. According to Castelli and Nizalova, the vast majority of infant mortality is avoidable [11]. Thus, it is important that governments continue to act to reduce the IMR further. Portugal has been one of the most successful countries worldwide in reducing IMR by introducing a series of healthcare system changes alongside socioeconomic improvements [8]. This reduction, greater in recent years, has been exceptional: IMR fell from 84.7 in 1960 to 2.8 in 2019 [12]. This compares favorably with the EU average of 3.5 in 2018 [13].

Vast improvements in social, environmental, and economic conditions contributed significantly to the reduction in IMR since 1960 [12,14,15]. These improvements included: environmental and sanitation infrastructure with improvements in basic sanitation and hygiene; educational policy measures to increase the literacy rate; social policies emphasizing maternal and child healthcare and family planning, including vertical mother-child health programs; and improvements in housing, diet, and overall quality of life, with a significant decrease in poverty rates [15]. It resulted in the narrowing of overall health disparities between more and less developed regions of the country [8,15,16].

Key policies and establishments have been introduced since 1989 which aimed to significantly restructure maternal care in Portugal with: reclassifications of hospitals into three different levels of care; designing of postgraduate studies in neonatology; and introduction of neonatal in-utero transport [17,18]. Owing to the aforementioned changes to maternity care, correspondingly the maternal mortality ratio (MMR) fell from 47.7 in 1974 [15] to 6.9 in 2016 [19].

In 2006, the National Commission on Maternal and Neonatal Health published a report entitled The National Program of Maternal and Neonatal Health, which evaluated access to maternal and perinatal care. It found that there were regional disparities in service organization and provision [20]. In response to the report, a policy aiming to provide safer and higher quality services, and to reduce caesarean section rates and overall system costs, by restructuring maternity units, was published [17,20,21]. This policy ordered the closure of public maternity units supporting less than 1500 births per year and the concentration of births into fewer and larger units. As a result, nine units were closed and the remaining maternity units were required to have at least two obstetricians, a neonatologist, a pediatrician, and an anesthesiologist present at all times to improve the quality of assistance for each delivery. Controversies over this restructuring sparked protests at the time of its implementation, from the general population, political actors and healthcare professionals [22]. However, the policy implementation was never evaluated, so there is currently a knowledge gap in what the impact of this reorganization has been on spatial inequalities and to what extent it influences avoidable infant mortality. The aim of this study is to analyze the impact of the 2006 policy of maternal and perinatal care reorganization on spatial inequalities in access to care and avoidable infant mortality.

## 2. Materials and Methods

Semi-structured qualitative interviews and surveys were carried out and thematically analyzed alongside the report by the National Commission for Maternal and Neonatal Health, the National Program for Maternal and Neonatal Health [20]. Different processes were used to recruit experts and mothers as participants. To recruit experts an established list of persons who were involved in the 2006 reorganization was used, and each person contacted individually by one of the authors. The sole inclusion criterion for expert participants was that they belonged to one of three categories: policymakers; healthcare workers; and researchers in maternal and perinatal health. To recruit mothers who have given birth in Portugal since the 2006, reorganization and preferably living in municipalities where a maternity unit was closed, social media was used. Mothers were identified using maternity- and infant-related Facebook groups. All 11 participants were emailed a survey specific to their role following their acceptance to participate.

Thematic analysis of the interview and survey responses in relation to the impact of the 2006 maternity unit closures on access to care and avoidable infant mortality revealed 4 main themes: impact of maternity unit closures; spatial access matters; current functioning of the healthcare system; and the medicalization of birth. The term “medicalization” was not used in any survey or interview questions but is used in this study to describe the view of birth as a pathological rather than a physiological process, and the subsequent increase in rates of medical intervention in the process, as outlined by Pintassilgo and Carvalho [23].

For anonymization purposes, each participant received an ID number including a letter which is used throughout the results section to refer to the specific participant (see Table 1 for anonymized participant details). The letter “P” denotes policymakers, “H” denotes the healthcare worker, “R” denotes the researcher and “M” denotes mothers. They responded to either a survey over email, or a semi-structured interview via a voice call, which was recorded and then transcribed using transcription software. See Supplementary Materials for surveys: Supplementary Materials S2 for survey sent to policymakers, Supplementary Materials S3 for survey sent to healthcare worker, Supplementary Materials S4 for survey sent to researcher and Supplementary Materials S5 for survey sent to mothers. The surveys and option to take part in an interview were open to participants in July and August 2020. One participant was a doctor as well as a mother, but completed the survey which was sent to mothers and responded as a mother. Ethical clearance was granted by Maastricht University (FHML/GH_2020.048) and written informed consent given by all participants; see Supplementary Materials S6 for the informed consent form. All participant information, consent forms and responses are kept on a password protected offline device.

To complement the results a quantitative spatial analysis was performed with Geographical Information Systems (GIS) methodologies using the software ArcGIS PRO (ESRI, Redlands, CA, USA).

First, data regarding the location of public maternity units was collected from the NHS portal [24] and private maternity units was collected from the birth advisor portal [25]. Locations of closed units were collected using news in the media. Data on birth rate, percentage of deliveries that did not happen in a hospital or at home (as a proxy of deliveries that may have happened on the road during transport to a maternity unit, but which includes all other deliveries outside of the home or hospital setting) and IMR by municipality and year were taken from the National Statistics Institute (Portuguese: Instituto Nacional de Estatística) [26]. Data was aggregated into three periods: 2001–2005 to evaluate the status before the policy was implemented; 2007–2011 to evaluate the short-term effects of the policy; and 2014–2018 for long-term effects. Therefore, short-term effects correspond to the comparison between the 2001–2005 period and 2007–2011 period, and long-term effects correspond to the comparison between periods 2001–2005 and 2014–2018. The evolution rate between different periods was ordered in three categories to allow better understanding: (1). decreased-municipalities where the evolution between periods was below 5%; (2). remained-municipalities where the evolution between two periods was between −5% and 5%; and (3). increased-municipalities where an increase higher than 5% was identified between two periods. The data was aggregated in this way and the cut-off of –5% and 5% were chosen by sensitivity analysis to counteract the effect of having an already low mortality rate on the results.

Second, geographic accessibility to the nearest public maternity unit by private car was measured by taking into account the location of maternity units and the road network managed through ArcGIS Online. Travel time for areas was measured in 10-min intervals below the threshold of 80 min.

## 3. Results

Results were organized according to the four themes which were derived from the qualitative analysis, alongside the findings and aims of the National Program for Maternal and Neonatal Health report and the subsequent policy change. See Supplementary Materials S1 “Table S1. Additional Quotes” for further quotes relevant to each section.

### 3.1. National Program for Maternal and Neonatal Health

In the report by the National Commission for Maternal and Neonatal Health, the National Program for Maternal and Neonatal Health [20], a variety of reasons were given for the substantial improvements in maternal and perinatal health and care over the last few decades. Broader factors included socio-economic improvement, investment in primary health care and national coverage of family doctors. Specific interventions included the implementation of the National Maternal and Child Health Program, which began in 1989. This involved reorganization of maternity resources with a focus on ensuring safe and quality care at birth.

Analysis of the current status of the maternal and perinatal health system revealed that coordination between different parts of the system was not optimal in all aspects, small maternity units which lacked the resources to ensure safe and quality care existed, and the workforce was aging while obstetrician training in recent years had been insufficient. There was an overall shortage of healthcare professionals in maternal and perinatal care and maldistribution of other resources among healthcare settings.

The Commission aimed to continue to ensure quality of monitoring of pregnant women and infants with equal access to care; ensure safe and quality care at a national level; and maintain and improve the health gains already achieved. Its future strategy centered around concentrating births into larger hospitals (seeing at least 1500 births per year) where quality and safe care could be guaranteed, without prejudice to the welfare or freedom of choice of the population. An additional goal was to reduce caesarean section rates.

This involved guaranteeing a quality transport system for pregnant women, accompanied by a specialist nurse, for distances of more than 20 km or 30 min travel time; enhancing distribution, training, and recruitment of human resources; identifying existing excellent practices and replicating them elsewhere; and guaranteeing physical resources and road access to another maternity unit before closing one.

All maternity hospitals were classified into one of four groups according to their ability to meet quality assurance requirements. Hospitals in each region were recommended to be closed or remain open based on its classification and local factors, such as geographic access, particularly regarding the construction of new highways throughout the country. Requirements for each level of hospital and healthcare setting were included that perinatal support hospitals were able to ensure the physical presence of an emergency team twenty-four hours a day (two obstetricians and one intern, one pediatrician, one anesthetist, and a team of nurses whose number and specialties are appropriate to the needs of the service).

Each Functional Coordinating Unit (UCF) for Maternal and Neonatal Health would consist of the hospitals and health centers in the area, with named coordinators for obstetrics, neonatology, maternal health, child health, and an overall coordinator. It would coordinate and evaluate the running of healthcare settings in its area, collect relevant statistical data and encourage epidemiological research to benefit care.

### 3.2. Impact of Maternity Unit Closures

The experts presented a positive overview of the closures and their impact. They stated that the decision to close some maternity units was correct and justified and has led to better and safer care for mothers and infants. The benefits of the policy are reported to have been particularly important for high-risk mothers and infants.

*“It is important for women and children to receive the best possible care, and that demands highly trained and specialized multidisciplinary teams; in a small country, with few resources, such as Portugal, it is important to organize those resources the best possible way.”*

(R5)

The impact on jobs was reported to be minimal, for those working in both closed and open units.

*“[Staff from closed units] were relocated in other units/services of the NHS.”*

(P4)

These benefits are reflected in the decline neonatal and infant mortality rates. The NMR (neonatal mortality rate) fell from 2.2 in 2005 to 1.9 in 2019, while the IMR fell from 4.3 in the period 2001–2005 to three in the period 2014–2018 [26].

Conversely, mothers did not see any clinical benefit from the closures and felt that they had been negatively impacted by them.

*“I don’t think that centralizing the services brought improvements on any levels [...] people have to spend hours driving in an emergency situation, or even for consultations, which is not recommended at all for pregnant women.”*

(M7)

Some mothers felt that the risk of delivering en route to the maternity unit, and therefore the risk of adverse outcomes, had increased following the closures.

**Figure 1:**
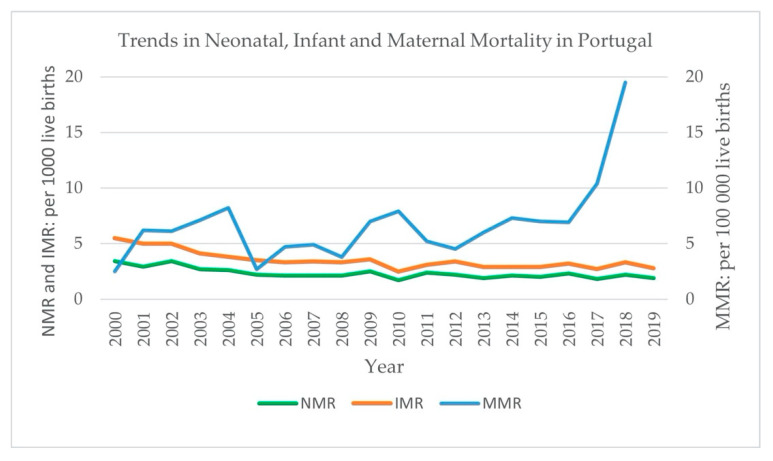
Trends in neonatal, infant and maternal mortality in Portugal from 2000 to 2019. NMR (neonatal mortality rate) and IMR (infant mortality rate) are calculated per 1000 live births, as shown on the primary vertical axis, and MMR (maternal mortality ratio) is calculated per 100,000 live births, as shown on the secondary vertical axis. Source: based on data retrieved from the National Institute of Statistics portal [26] and Pordata [19].

Statistical data show that infant and neonatal mortality have both continued to decrease since the closures in 2006 while maternal mortality has fluctuated with time (Figure 1). The reason for the differing trend in maternal mortality and its sudden increase in recent years is unclear.

### 3.3. Spatial Access Matters

**Figure 2:**
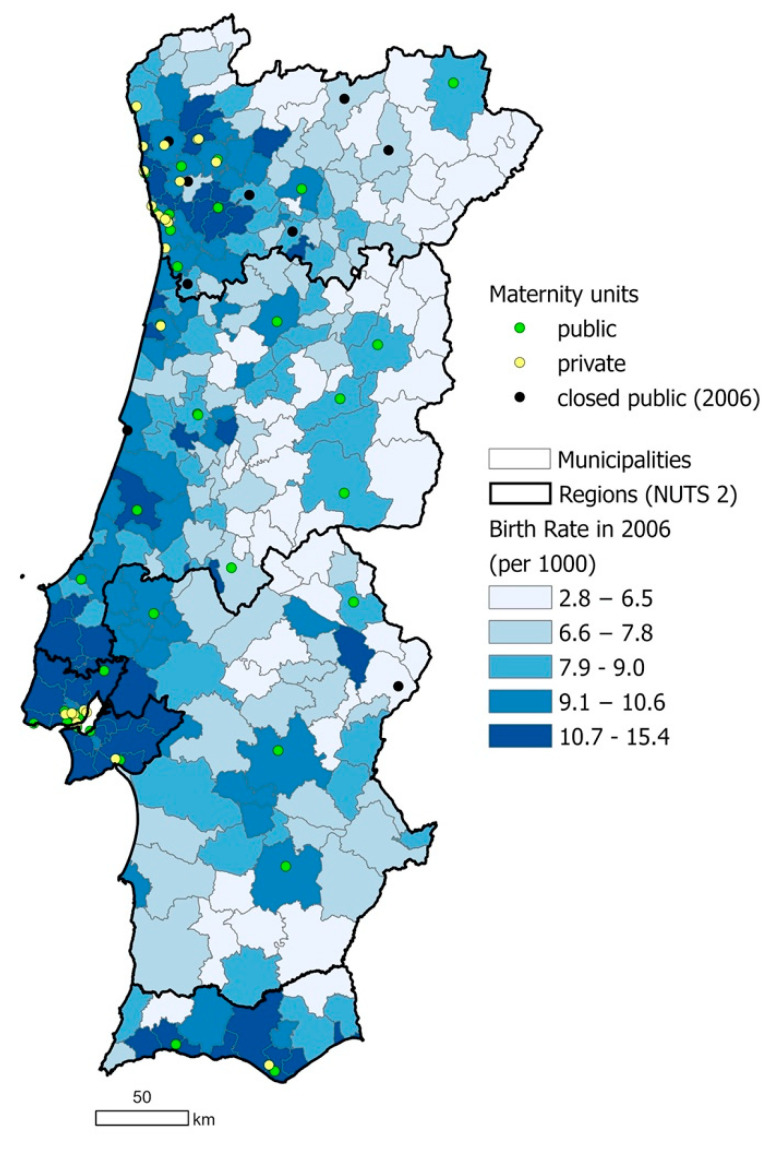
Location map of running and closed public maternity units and running private maternity units, as well as birth rate by municipality, in Portugal in 2006. Source: based on data retrieved from the National Institute of Statistics portal [26], the NHS portal [24] and Birth Advisor portal [25].

The analysis of spatial access to maternity units revealed that women living inland and further away from major cities were negatively impacted by the 2006 public maternity unit closures. After the reform mainly maternity units in the hinterland were closed which leads to spatial inequalities in access to care. This is visualized in Figure 2.

**Figure 3:**
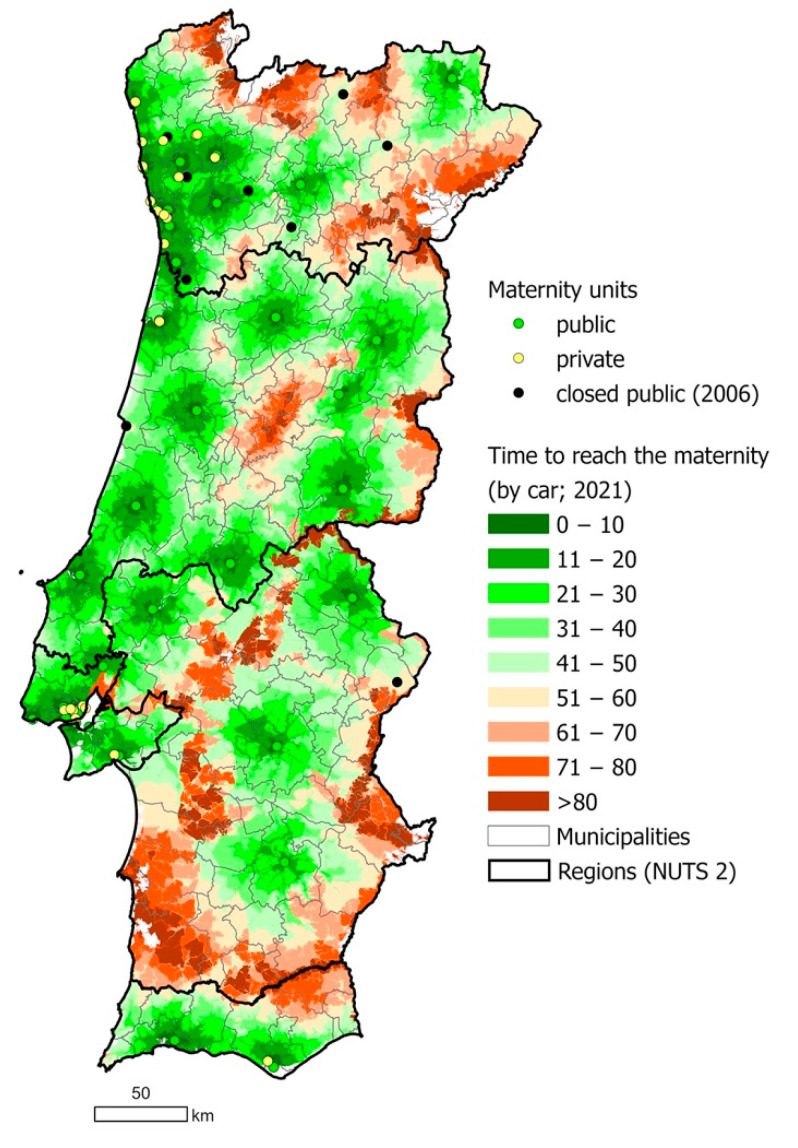
Map showing travel time by car to reach the nearest maternity unit, municipality boundaries, and locations of running and closed (in 2006) public maternity units and running private maternity units, in Portugal in 2021. Source: based on data retrieved from ESRI, the NHS portal [24] and the Birth Advisor portal [25].

Geographic accessibility to public maternity units in Portugal by car was analyzed. Figure 3, a map showing the time needed to reach the nearest maternity unit by car throughout Portugal, shows disparities between different areas and municipalities. Some municipalities which contained a public maternity unit before the 2006 closures, no longer did following the closures. Many areas within these municipalities, and even the total area of some, required more than 50 min of travel time by car to the nearest unit following the closures. Certain areas face greater inequalities than others; Alentejo region and rural areas located near the border with Spain require much more time for residents to reach the nearest maternity unit than other parts of the country.

Mothers living further from remaining maternity units in this study reported these negative impacts. Women who previously could have attended a maternity unit in their own town were now a great distance from their nearest maternity unit, which was reported to have had a negative impact on their wellbeing.

*“Being an hour away from the nearest maternity did cause some extra anxiety.”*

(M7)

The experience of giving birth in a hospital different to the one where their prenatal scans and consultations were carried out was also negatively perceived, although the impact was minimized in some cases by visits to the unit to familiarize themselves with the environment.

*“Having appointments in a hospital where you don’t give birth is not the best.”*

(M3)

Road connections were a recurring theme and rated as an extremely important factor in enabling the centralized system to function well. Sufficient transport networks to the larger, open maternity units were criteria for implementing the closure of a smaller unit. These units were therefore not closed until works including improved roads had been completed.

*“The later ones [closures] it was [...] to wait for the new highway [...] we only finish [the closure] when we can [...] guarantee that [...] travel can be done in a very short period.”*

(P6)

Unfortunately, despite the improvements in road connections in many parts of Portugal, mothers from areas which have not had such improvements yet and with poor road links to maternity units may still face reduced access to care, as acknowledged by both a mother (M2) and a healthcare worker (H2) in this study. The closure of smaller maternities has therefore had a much greater impact on mothers living in areas with poorer road links to maternities, who face spatial inequalities in access to care; during emergencies this comes with an increased risk of delivering in transport.

*“I consider myself lucky to have quick access to the maternities in the region, the same I cannot say about people who live in more remote places and with more complicated connections, in which many mothers end up having babies in ambulances making the birth delivery experience far more traumatic.”*

(M2)

**Figure 4:**
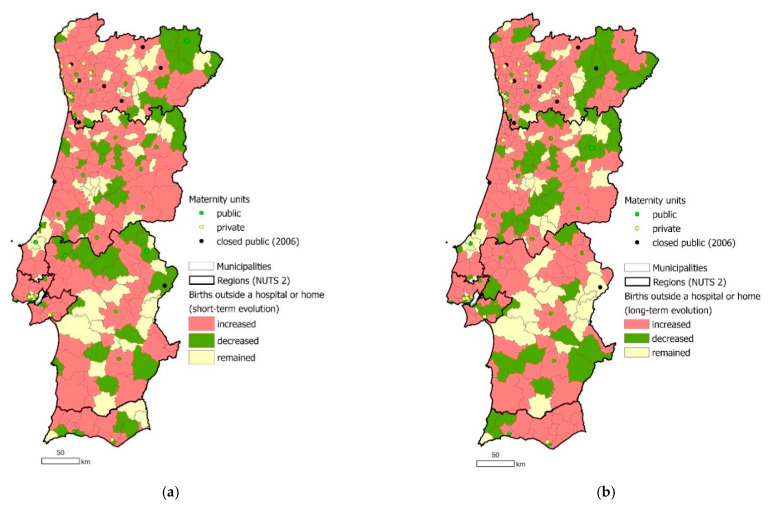
Impact of 2006 policy change: change in roadside deliveries in Portugal in the short term (**a**) and long term (**b**) compared to the closure of maternity units. Source: based on data retrieved from the National Institute of Statistics portal [26], the NHS portal [24] and the Birth Advisor portal [25].

There was an overall small long-term increase in the percentage of births which likely occurred during transport, as they did not happen at hospital (in a maternity unit) or at home, in each municipality (Figure 4); from 0.1% to 0.4% between the first and last period of the analysis. Some municipalities closer to a maternity unit which was closed showed this increase, reflecting the fears of mothers in this study of giving birth during transport. However, an increase was also evident in municipalities which were unaffected by the 2006 closures; therefore, there are likely to be factors other than the restructuring which account for this.

Even in low-risk pregnancies, mothers who live at a greater distance from the maternity unit where they give birth experience different care to those who live closer to the unit. The distance from specialized services was seen as an issue for mothers in high-risk pregnancies at all stages of the pregnancy. Three mothers (M3, M7, and M8) reported having frequent scans and consultations due to having a high-risk pregnancy, existing medical condition, or previous difficulties, at units up to 50 km away. Therefore, these mothers had to frequently travel a much greater distance which involved higher stress, greater costs, and more time. This continued into the postnatal period for mothers and babies requiring hospital-based care rather than primary-based care. The extra costs and burden faced by such mothers is another way in which their welfare has been impacted negatively, and the Commission has failed to achieve its goal for all mothers.

*“Pregnant women, not residing in the same place where the maternity is located, are admitted several days before the estimated delivery time [...] their stay in hospital/maternity is precautionarily ‘stretched’, before and after delivery.”*

(P4)

**Figure 5:**
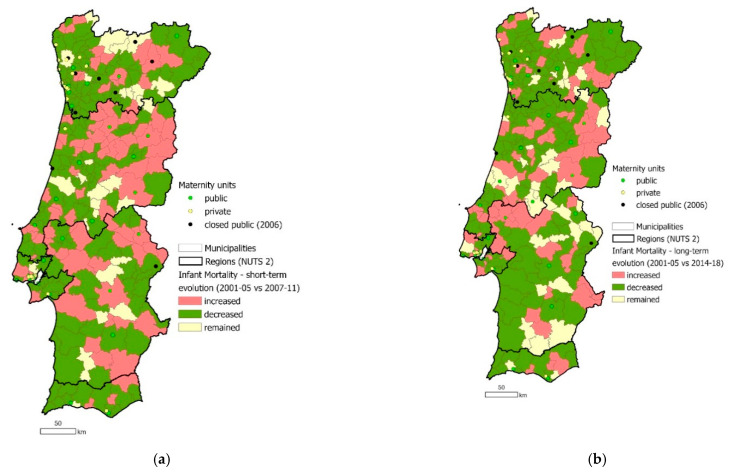
Impact of 2006 policy change on infant mortality (%) by municipality in Portugal in the short term (**a**) and long term (**b**). Source: based on data retrieved from the National Institute of Statistics portal [26], the NHS portal [24] and the Birth Advisor portal [25].

The Commission’s overarching goal was to improve and guarantee safety and quality of care for all mothers and babies. One metric which may reflect the achievement of this goal is the reduction in IMR. Figure 5 shows that while some municipalities closer to a closed maternity unit faced a short-term increase in IMR, this increase was not evident in the long term; the IMR in Portugal had an overall long-term decrease from 4.3 to 3.0%. In addition to IMR, the Commission also considered spatial access to maternity units when planning how to best guarantee safe and quality care. For example, when deciding whether a maternity unit should remain open or closed, geographic access of local populations to the nearest maternity unit was considered as well as births per year; in cases where units were closed following new highways which would soon enable local populations to access further units, the closures were not implemented until the new highways had been completed and opened, to ensure access was preserved.

### 3.4. Medicalization of Birth

Neglect of the father during the birth was a key factor for many women in their inability to access the full care and support they wanted. They found that the father was ignored by healthcare professionals or given very little support and was often not able to be present for a variety of reasons. This impacted freedom of choice of the mothers, particularly as different mothers reported having access to different treatment by healthcare professionals.

*“When my delivery turned medical, [the father] was initially not allowed to follow me to the intervention room [...] in which case he would not have been able to see his child born. Luckily, we managed to convince the doctors to make an exception (based on the fact that we are both doctors ourselves, so this felt a bit unfair later on to others who would not have this ‘negotiation’ room).”*

(M4)

Guaranteed inclusion of the father during the birth, an aim of the Commission, was not perceived to be fulfilled for participants, despite its emphasis both by a policymaker and throughout the report.

*“[An aim of the policy was] nationally to guarantee [...] that the father can be [...] with their wife.”*

(P6)

One non-native mother (M4) compared her experiences of birth in Portugal to her experiences in other countries and found that the nature of birth in Portugal is highly medicalized with significant levels of institutionalization and intervention. She and others (M2, M3, M4, M6, and M8) expressed a wish for a more holistic view of birth and for maternal and perinatal care to be delivered accordingly.

*“A big improvement [...] would be if birth was considered less a ‘medical event’ and more a ‘natural process’, where the woman giving birth would be considered a mother rather than a patient”*

(M4)

These high levels of intervention are reflected in the caesarean section rates in Portugal, which have remained high since the maternity unit closures: they were 34.7% in 2005, peaking at 37.7% in 2009, and only lowering to 33.1% in 2017 [26]. This is despite the creation of the National Commission for the Reduction of the C-Section Rate in 2013, which aims to ensure medical justification of c-sections [21].

### 3.5. Current Functioning of the Healthcare System

The free-of-charge nature of the Portuguese NHS was stated as a key strength of the current maternal and perinatal healthcare system. Health expenditure as a percentage of GDP has remained steady over the last five years at an average of 9.4% In 2018, 66.5% of expenditure was public and 33.5% was private [26].

Communication between primary and secondary care within the public healthcare system was also highlighted as a strength. In maternal and perinatal care, Functional Coordinating Units link the primary and hospital care of pregnant women, and perinatal support units are linked to differentiated perinatal support units, which provide more specialized care for more vulnerable infants, in order to coordinate the care of women with high-risk pregnancies.

*“The strengths of maternal and perinatal healthcare system in Portugal are related to the close cooperation/care integration between hospital (pediatric, maternal/obstetric and perinatal referral departments) and primary care centers (family medicine practice), through [...] functional coordinating units.”*

(P4)

However, this communication may be disrupted by the fact that many mothers reported accessing both private and public care during their pregnancy, which relies on the mothers to transport their own medical information and examination results between different hospitals.

*“My obstetrician provided me with all the exams and data that I easily took with me.”*

(M2)

An inequality in access to care that was evident in the mothers’ responses was the availability of freedom of choice in all aspects of care. Freedom of choice was highlighted throughout the Commission’s report; however, awareness and accessibility of choice varied widely amongst the mothers. Some mothers felt that they were provided with sufficient information and the ability to choose where they would give birth, while others reported being given insufficient information and not allowed any choice in their care.

*“The doctor [...] explained [...] that I was free to choose any hospital I wanted and that she would refer me to any other place.”*

(M4)

*“I wasn’t given enough information, for the most part I was spoken to and never felt really listened to.”*

(M6)

For those that declared to have had freedom of choice, they decided to access private healthcare in at least one aspect of their pregnancy and delivery in order to fulfil their choice. Most private maternity units were located in coastal, metropolitan areas in 2006 (Figure 2). For mothers who had the financial capacity to access private care, only those in metropolitan areas would therefore be able to act on their choice of location of care. Needing to utilize private healthcare in order to access women’s choices compounds the inequalities in access to freedom of choice, as not all mothers may be able to afford private care.

*“I occasionally consulted a private doctor because I wanted the birth delivery to be in [another hospital] and not in [the automatically provided hospital]”*.

(M2)

Accessing private care was also reported by one mother (M4) to be more likely to result in interventions during birth. These interventions included caesarean sections. Higher rates of caesarean sections are a contradiction to the aims of the Commission.

*“I [...] know of quite a few examples of births being induced or [caesarean] sections being performed because of ‘logistic’ rather than medical reasons (eg., let’s convert to a section because it’s Friday afternoon).”*

(M4)

Responses relating to the current functioning of the healthcare system contained two key factors: first, that of an ageing and stretched workforce; second, the increase in maternal age and the higher risks and costs that accompany it.

*“Demand pressure and wearing of human resources has compromised readiness and quality of responses. There is presently, and in the foreseeable future a problem not only of progressively scarce human resources, but also of aging of existing human resources.”*

(H2)

*“Maternal age has been increasing steadily in Portugal, during the last decade(s) and it is associated [with] the increase of fetal/perinatal and maternal risk.”*

(P4)

**Figure 6:**
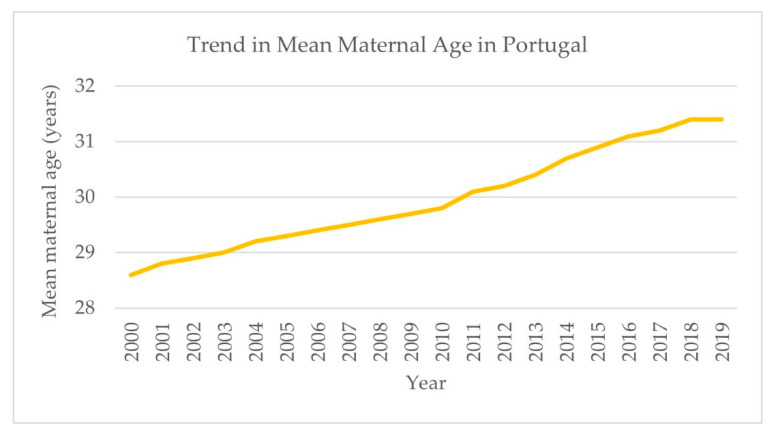
Graph showing trend in mean maternal age at birth of a child in Portugal between 2000 and 2019. Source: based on data retrieved from the National Institute of Statistics portal [26].

These aspects were noted as challenges to both the current and future functioning of the healthcare system in general and the maternal and perinatal care system. Increasing maternal age in Portugal is evident in statistical data (Figure 6).

## 4. Discussion

Overall, the closure of smaller maternity units as part of the 2006 restructuring of maternal and perinatal care in Portugal was successful in its aims according to participants: safe and high-quality care was improved across all maternity units for all women and newborns. An overall decrease in an already extremely low IMR was achieved between 2001 and 2018, continuing the successful trend of reducing IMR greatly over the last few decades. However, despite initial considerations of spatial accessibility by the Commission, inequalities in geographic access to maternal and perinatal care persist, as revealed by the participants in this study. Access to maternity care must be equitable in addition to care being of high quality in order to ensure good outcomes for mothers and infants [27].

The guarantee of care was intended to be provided without prejudice to the welfare or freedom of choice of the population. Results state that this intention was not fully realized as mothers may face greater anxiety and fear due to distance and giving birth in a different hospital to the one where they accessed prenatal care. Mitigating procedures can place additional costs and burdens on both mothers and the healthcare system. These burdens may further restrict mothers from accessing wider care on a more regular basis, such as labor and motherhood classes.

These inequalities are particularly important given the demographics of Portugal’s population: while the proportion of the population living in urban areas is increasing, 34% of the population were living in rural areas as of 2019 [28]. In 2011, women aged 15–49 years old represented 37.4% of the female population living in predominantly rural parishes [26]. Maternity units are concentrated in coastal and urban areas. Therefore, a significant proportion of mothers live far from maternity units and are more likely to face spatial barriers to healthcare services (see Figure 2 and Figure 3) [29]. The impacts of these inequalities are compounded by the spatial maldistribution of healthcare professionals in Portugal, with high regional differences in the number of physicians per 1000 inhabitants; in 2011, the Northern and Lisbon/Vale do Tejo regions had 74% of the country’s physicians for 65% of the population, while the Central, Alentejo, and Algarve regions had 18%, 4%, and 4% of physicians for 23%, 7.5%, and 4.5%, of the population respectively [30]. The demand and pressure of human resources is related to an aging workforce and the overall shortage of human resources, which remains a major obstacle to the optimal functioning of the NHS [29,31]. A recent study revealed that the 2008 economic crisis and austerity measures were perceived to have aggravated the deficiency in human resources in Portugal [32,33]. Confirming this, a study in 2016 reported that many medical doctors declared that they would opt to migrate due to a changed working environment induced by the crisis and austerity measures [34].

Regional and spatial inequalities in access to healthcare services are a key issue in Portugal [2]. Nationally, large disparities between municipalities exist in distance from hospitals, with isolated communities, those with a higher share of the older population and those located closer to the border with Spain facing greater disadvantages [35]. Regional disparities in rates of avoidable mortality have been previously found, with the Algarve, Alentejo, and Lisbon and Vale do Tejo regions faring worse than the Central and North regions [20]. The concentration of maternity services along the coast correlates with population density and birth rates, which are much higher in urban areas along the coast than rural areas inland [21]. However, it also demonstrates the potential for spatial inequalities in access to care. While the Commission considered geographic access by road when planning the maternity unit closures, pregnant women living inland may still live up to 90 km from the nearest maternity unit with a travel time of over one hour, a distance not faced by women living along the coast [17].

In European countries with parallel public and private healthcare systems, the private sector is more frequently accessed by the more affluent population [29,36]. Within the EU, Portugal holds a relatively high percentage of out-of-pocket health expenditure; in 2017, it accounted for 27.5% of total health expenditure in Portugal but 21.5% in the EU on average [37,38]. In Portugal, women who accessed only public prenatal care were found to be more likely to be less well educated, unemployed, in unskilled occupations or to have a lower income than those who accessed private care [7]. The results of this study suggest that some women who have the economic ability utilize private care in order to maintain their freedom of choice of place and type of maternal and perinatal care. This may be influenced by the fact that prenatal scans are performed more frequently in private care, which is reassuring for parents, as well as private care allowing greater flexibility and specificity in appointment times. Private facilities may additionally offer better options for attendance and inclusion of fathers at birth. The closure of public maternity units may have further contributed to mothers choosing private care, if they felt that their access to public care was restricted. This factor increases spatial and economic inequalities in access to care, as not all women can necessarily afford private care, and those that can suffer an increased economic burden when accessing their chosen care. As results showed that private maternity units were concentrated in coastal and metropolitan areas, those living more remotely may have been also impacted by spatial inequalities in access.

The presence of high-quality road transport and a strong road network were important factors in this study in ensuring sufficient functioning of the regionalized maternal and perinatal care system for experts and sufficient access to care for mothers. These transport and network aspects have been central parts of the maternal and perinatal care system in Portugal since 1987, when the Neonatal Transport System was first introduced, and are now fundamental to the regionalized system. Emergency transport is provided for mothers from home to hospital during labor and for neonates between hospitals when required [30]. Results demonstrate the change in rates of births that did not happen in the hospital or at home, with a small increase overall between 2001 and 2018, highlighting its importance.

Furthermore, due to legislation home births are only available in Portugal through private healthcare [39], which widens economic inequality of access to a woman’s chosen care during birth. One study found that home births, as well as more “alternative” forms of care, were more common among women of a higher social class, with a higher level of education and more resources [23]. Until 1961, 80% of births in Portugal were home births, a trend which continued into the 1970s. These births were usually attended by women with no formal medical training, as opposed to trained midwives [40]. The restriction on home births came about due to poor outcomes resulting from home births in the past. While the initial restriction on home births and move towards hospital births was necessary to improve outcomes for mothers and infants, the current situation is flawed: home births in Portugal now exist in a “legal void” [41], with no official network or referral system for nurse-midwives. Consequently, women who would prefer a home birth may face heightened risks, such as their nearest midwives being extremely far away when they go into labor [39], increasing the risk of avoidable infant mortality.

The lack of home birth as an option for public healthcare users is one factor in the highly medicalized state of pregnancy and birth in Portugal, and the movement towards this medicalized state has been well documented [35,39]. In 2010 and 2011, 99% of births in Portugal took place in a hospital [23]. The presence of doctors at birth has been increasing while the presence of obstetric and non-obstetric nurses has been decreasing. There is a trend for scheduling births on weekdays, with rates of instrumental deliveries and caesarean sections increasing until recently and remaining high [20]: episiotomy rates for vaginal birth were 72.9% in 2013 [42]. This movement is even more evident in the private sector: the rate of caesarean sections more than twice as high in private hospitals as public ones [23]. The greater number of private hospitals in the North region (as shown in Figure 2) correlates with the highest rate of caesarean sections occurring in the North: 36% in 2018 (the lowest was the Central region with 27%) [26]. The frequent use of private care may be of concern, as unnecessary medical intervention can lead to poor outcomes for the mother and their infant [43]. As it also contradicts one aim of the Commission in implementing the 2006 policy in order to reduce the rates of caesarean sections, it highlights the need for further action on this issue.

Other studies have also emphasized calls for birth to become more “humanized” and for women to be empowered in the process [42]. There are currently limited options in Portugal for holistic or alternative maternity care, including arrangements such as water births, as well as home births, in the public sphere, despite requests for such options from the public [44]. Combined with the lack of an official, public network of qualified midwives to assist home births, this presents huge risks to mothers who choose to give birth at home, and their infants. Therefore, excluding holistic care and home births from the public sector may increase stress for the mother and risks for both mothers and infants. This is contradictory to the aims of the Commission in restructuring the maternal and perinatal care system to guarantee safe, quality care for all mothers. The experiences of mothers in this study and the findings of other studies [39,42] indicate that women’s autonomy is often not upheld during birth. Additionally, the exclusion of the father from the birth experience is likely to increase stress for both parents [45] and consequently increase the healthcare and economic burdens on the mother and the NHS. Other healthcare systems can be looked to for examples of more holistically integrated maternal and perinatal care. In other countries, midwives have a central role in maternity care, birth is viewed physiologically rather than as a pathological process, and primary care practitioners have a much greater role in pregnancy and birth care [39,46,47].

The Portuguese national effort to reduce caesarean section rates following 2009 had some success, with a small decrease from 36.6% to 33.1% over five years [20]. There is significant variation in caesarean section rates within the EU: the average in 2014 was 27.5% [48], while in 2017, the highest rates were experienced by Cyprus with 54.8%, and the lowest by Finland with 16.5% [49]. The reasons for a higher preference for caesarean sections by mothers and healthcare professionals in Portugal must be further researched and understood. This will aid understanding of why caesarean section rates remain high in Portugal and provide insights on possible ways to continuously reduce them. A key factor which must be considered is that maternal age in Portugal is increasing in line with other European countries [50]. Higher maternal age is associated with poorer outcomes for both the mother (e.g., postpartum hemorrhage and eclampsia) and the infant (e.g., preterm birth and low birth weight) [51]. With increasing maternal age, the incidence of high-risk pregnancies increases, and with it the burden on specialized care and obstetricians. This may lead to a higher likelihood of having a caesarean section. With an already stretched workforce, this may further increase inequalities in access to care. This is principally important given that maternal mortality has not followed the same decreasing trend as infant and neonatal mortality and increased significantly in 2018. Seventeen women died due to complications during pregnancy, childbirth, or the puerperium, causing the MMR (maternal mortality ratio) to increase to 19.5, a large jump from 10.4 in 2017. Of these 17, 15 died in a hospital or clinic, including both NHS and private settings, 1 died “at home” and 1 died “elsewhere”, meaning not in a hospital, clinic, or home. The Director-General for Health was reportedly “concerned” at the time [52].

### Strengths and Limitations

While Portugal is the focus of this study, the findings will be of importance for other countries; in particular, countries with similarly structured or publicly tax-funded healthcare systems, countries with spatial disparities in access to care, or those looking to restructure the maternal and perinatal care system.

The main limitation of this study was the small sample size of interviewed participants. Consistent themes were evident in all responses, suggesting that the identified themes are important to the wider population, and a variety of opinions were presented which were often held by more than one participant. The use of snowball sampling may have introduced bias. Further limitations were restricted travel constraints and in-person open interviews which resulted in the use of surveys as the main method of data collection. Further data availability on population health indicators such as roadside deliveries, caesarean section variations, maternity consultations in private, public, and primary care, and average out-of-pocket payments for maternity care could have strengthened the results. In particular, granulated small-scale data is missing for small scale analysis which is required to enhance studies on overall regional inequality.

## 5. Recommendations

In order to reduce inequalities in access to care and ensure women’s freedom of choice during pregnancy and labor, a holistic, woman-centered approach to birth could be adopted in the public healthcare system. Reducing spatial inequalities in access to care and avoidable infant mortality in Portugal requires all women to have sufficient access to their required and desired healthcare facilities. Maternity units should be targeted with teams specific to their situation and clinical requirements in order to improve their capacity. The criteria for open and closed maternity units could be reviewed, since in the years since the 2006 report, medical knowledge and service requirements may have changed. Finally, since an official study on the impact of the 2006 policy has never been done, a technical analysis of its outcomes and further improvements would enhance understanding of policy effectiveness.

Communication between the health ministry and health service users, such as mothers, could be evaluated for improvement, particularly with regards to mechanisms implemented to ensure their safety and improve the quality of their care, in order to improve confidence in the health system. Communication between primary and hospital care, and public and private care, could also be reviewed to ensure efficient running of the health system. As municipalities are responsible for local primary care activities such as mother and child health care plans, local administration should investigate the barriers to adequate and equitable access to primary care for mothers in each municipality. They should further identify contextual or social determinants which may contribute to avoidable infant mortality.

Future research in Portugal would be beneficial in areas such as the medicalized status of pregnancy and birth; geographic factors in access to maternity care; human resource distribution and quality of service response; out-of-pocket payments for maternal and perinatal care; comparing the usage and IMR of public and private maternal and perinatal care; reasons for use of caesarean sections; and maternal mortality causes and rates. Increased data availability for these areas and higher quality data at local levels in all areas would further the quality and use of such research. Local data availability and quality is particularly relevant for research on, and policy changes tackling, healthcare inequalities and would benefit from further investigation.

## 6. Conclusions

The outcomes of this study will help to further inform policymakers, managers, and researchers in both maternal and perinatal healthcare and general health system restructuring.

Spatial inequalities in access to maternal and perinatal care persist in Portugal, which can lead to economic and welfare inequalities. Since the maternity unit reorganization in 2006, avoidable infant mortality per 1000 live births has decreased from 4.3 in the period 2001–2005 to 3 in the period 2014–2018 [26]. Despite great success in the reduction of infant mortality, spatial access to maternal and perinatal care should be reflected on to further enhance equity of access to care.

The currently highly medicalized maternal health care system would benefit from including: further outpatient and home-based maternity care; home births supported by the NHS; the introduction of a midwifery system; greater involvement of non-specialist professionals; and involvement of the father/partner throughout the intrapartum period, with the option to be included during the birth. This, along with other changes outlined, will help to improve perceived quality of care, and to evaluate and reduce inequalities in access to care and reduce avoidable infant mortality.

## Figures and Tables

**Table 1:** Participant Details.

ID	Participant Group	Type of Response	Supplementary Materials	Location	Sex (M/F)	Year of Birth (S)	Affected by Closures
P2	Policymaker	Survey	S2	Central	M		
P4	Policymaker	Survey	S2	Central	M		
P6	Policymaker	Interview	S2	North	M		
H2	Healthcare Worker	Survey	S3	Central	M		
R5	Researcher	Survey	S4	Unknown	F		
M7	Mother	Survey	S5	Central, Urban		2015, 2018	Yes
M3	Mother	Survey	S5	Central, Urban		2020	Yes
M2	Mother	Survey	S5	North, Urban		2014	Yes
M4	Mother	Survey	S5	Central, Urban		2019	Unknown
M6	Mother	Survey	S5	Central, Urban		2017	No
M8	Mother	Survey	S5	Central, Rural		2011, 2018	Yes

## Data Availability

Qualitative interview data is not made available due to privacy concerns.

## Supplementary Materials

The following are available online at https://www.mdpi.com/1660-4601/18/3/1242/s1, Supplementary Material S1: Table S1. Additional Quotes, Supplementary Material S2: Survey Sent to Policymakers, Supplementary Material S3: Survey Sent to Healthcare Worker, Supplementary Material S4: Survey Sent to Researcher, Supplementary Material S5: Survey Sent to Mothers, Supplementary Material S6: Participant Information and Informed Consent Form.
