# Supplementary material for: Spatial Access Matters: An Analysis of Policy Change and Its Effects on Avoidable Infant Mortality in Portugal
Source: Int J Environ Res Public Health. 2021 Jan 30;18(3):1242. doi: 10.3390/ijerph18031242 (PMC7908299; doi:10.3390/ijerph18031242)
Supplement: Supplementary file 1 [file ijerph-18-01242-s001.pdf]

<u>Supplementary Material</u>

<u>Supplementary Material 1 (S1)</u>

Table S1. Additional Quotes

Theme	Quotes
**Impact of Maternity Unit Closures**	*"It is hard to justify the expense of permanent obstetric emergency teams...in hospitals with very few deliveries and distancing few kilometres from each other. On the other hand, human resources are scarce and must maintain a certain degree of...skill, through constant practice. I think the principles for selected closures were correct" (H2)*<br><br>*"The reason for the 1500 births [cut-off] was the necessity of preserving the health professionals' technical capability, through regular exercise of some essential practices" (P2)*<br><br>*"The closure of those maternities was, from my point of view, a public health issue, not only due to the likely negative health impact of their functioning (quality and safety of obstetric and neonatal care), but also because of their economic impact (costs and NHS sustainability)" (P4)*<br><br>*"Pregnant women at risk could [following the closures] give birth surrounded by better teams in number and technical training and newborns could be assisted with all the needed pre, peri and postnatal emergency response capability and with immediate stabilization at the place of birth" (P2)*<br><br>*"It is epidemiologically plausible that avoidable infant mortality has been reduced due to the closure, in 2006, of small-sized maternities/obstetric units" (P4)*<br><br>*"[the closures] allowed women to be cared for and to have their babies in units with highly specialized teams, which contributes to lesser problems and very low numbers in maternal morbidity...since primary health care has assured most of the necessary care in normal pregnancies, access is equal" (R5)*<br><br>*"The closing of [a nearby unit] did not signify an extra load of work to our unit" (H2)*

	"The allocation of human resources was revised, and professionals from closed units were transferred to the receiving services" (P2)<br><br>*"I honestly don't understand this closure. Speaking of [a now closed maternity], we had excellent conditions, teams well prepared so the closing was unreasonable" (M2)*
**Spatial Access Matters**	*"Until 2006 [my city] had a maternity ward, with excellent conditions, which was closed…[now] we have two maternity hospitals in the region...at 66km and...58km" (M2)*<br><br>*"I was about 50km far from the nearest open maternity unit. Whereas before the closure I had a maternity in the city I live in, and that is about 5 minutes away from home" (M7)*<br><br>*"The nearest maternity unit is about 40km away from my home...I was closer (around 20km) to one of the closed maternity units" (M8)*<br><br>*"I...went to the maternity unit once to visit and get to know the premises" (M7)*<br><br>*"Portugal has, since the 1990s, a good road network and highways have improved geographic access to major health/hospital facilities" (P4)*<br><br>*"luckily with the...motorway it is possible to travel to either [one of the nearest units] or [another of the nearest units] in approximately 30 minutes" (M2)*<br><br>*"We always have had, not so rarely, cases of women having their children during transport: those coming to [a major city] from municipalities more distanced to the interior, with poorer road access" (H2)*<br><br>*"For pregnancies in need of specialized care there may be more difficulties, since referral maternities are usually placed in the main cities, so more distant for populations living in rural areas" (R5)*
**Medicalization of Birth**	*"The post-natal system is strange to me...being...raised in a context where home deliveries are normal and hospital deliveries usually lead to same-day discharge" (M4)*

	"Births should be more humanized. There are still very archaic practices [unspecified] carried out in many places according to testimonies I heard" (M2)<br><br>*"There is no room for the fathers to wait nor a bathroom on the inside of the building. They have to, sometimes sleep in the cars until a call is made for them to go and be part of the birth and if it is a c-section they are not allowed" (M3)*<br><br>*"Could do better in supporting mother and baby post-birth, allowing father or other companion to stay more hours" (M6)*<br><br>*"I only missed some attention to the mental health of the pregnant/mother, I consider that the health professionals paid more attention to the biophysical aspects whereas the mental health dimension was a little dismissed" (M8)*<br><br>*"It could improve in terms of mental health coverage – there are few professionals and few specific services, either for women/couples during their pregnancy and transition to parenthood as for children and adolescents" (R5)*
**Current Functioning of the Healthcare System**	*"Strengths:...free access (to contraception and care during labor, delivery and puerperium)" (H2)*<br><br>*"The expertise of healthcare professionals is high, there is generally a large trust in the obstetric system and people are proud of the low perinatal mortality rates in the country" (M4)*<br><br>*"Women can always choose the place...for the birth...for us it was a very important detail...so, so important" (P6)*<br><br>*"I had no problem with my choice, since my obstetrician provided me with all the exams and data that I easily took with me...However, I know that there are obstetricians who do not have the same attitude as the one who accompanied me" (M2)*<br><br>*"For my second pregnancy, the scans were done at a private doctor and were done monthly. This is much more reassuring" (M7)*

Supplementary Material 2 (S2)

Survey Sent to Policymakers

1. What are the strengths of the maternal and perinatal healthcare system in Portugal today, and what improvements could be made?

2. What roles have you had in policymaking in maternal and perinatal healthcare in Portugal? Were you involved in the 2006 restructuring of the maternal and perinatal healthcare system, and the resulting closures of some maternity units? If so, please describe your role.

3. Geographical or road and transport reasons are noted as reasons to keep maternity units open which would otherwise have been closed due to seeing fewer than 1500 births per year. Could you elaborate on what these reasons were specifically?

4. Why or how was 1500 selected as the number of births required for sufficient quality of care? Why or how was the cut-off of 20km distance or 30 minutes transport time selected for transporting pregnant women?

5. In cases where units remained open for geographical reasons, did this lead to any compromises on quality or safety of care? What steps were taken to ensure sufficient quality?

6. What interventions were implemented to help the maternity services receiving mothers from closed units and the local emergency transport units?

7. What interventions were implemented to counterbalance the loss of access to care for mothers affected by the closures?

8. What happened to the health personnel and resources from the closed units?

9. Do you think that the closures had any impacts on pregnant women's access to care during pregnancy and birth, whether positive or negative, particularly in terms of their geographical access?

10. What impact did the 2006 closures have on the survival of particularly vulnerable babies, such as pre-term, very pre-term and very low birth weight babies?

11. Would you change anything about the policy which was implemented at the time, and why?

12. Do you believe that the 2006 restructuring and maternity unit closures reduced avoidable infant mortality? Why? Is there a study providing evidence for this?

13. Please add any further comments that you'd like to make:

<u>Supplementary Material 3 (S3)</u>

Survey Sent to Healthcare Worker

1. What are the strengths of the maternal and perinatal healthcare system in Portugal today, and what improvements could be made?

2. At the time of the maternity unit closures in 2006, what was your occupation, and which hospital did you work in?

3. Was your job or your place of work affected by the closures? How? Were the effects positive or negative?

4. If your unit remained open, were there any changes made? If it was closed, where were the staff and resources relocated to?

5. Following the closures, roughly what proportion of women lived close enough to the hospital to organise their own transport when in labour, and how many made use of the transport system?

6. How well did the transport system function? Did women arrive in good time before giving birth? Did women ever arrive having already given birth?

7. Did you know of any mothers choosing to move home in order to live closer to a maternity unit before giving birth?

8. Were there any changes to training in maternal and perinatal health for healthcare workers following the 2006 report? What were these changes?

9. Did you notice any change in the level of communication between different groups and institutions in the healthcare system following the 2006 restructuring? Did you feel that communication improved?

10. Would you change anything about the policy which was implemented at the time, and why?

11. Please add any further comments that you'd like to make:

Supplementary Material 4 (S4)

Survey Sent to Researcher

1. What are the strengths of the maternal and perinatal healthcare system in Portugal today, and what improvements could be made?

2. What is the focus, or what has been the focus in the past, of your research in maternal and perinatal health in Portugal?

3. What have been the key factors in reducing neonatal, infant and maternal mortality in Portugal over the last 50 years?

4. Is it possible to differentiate between avoidable and unavoidable causes of infant mortality based on the way that infant deaths are officially recorded?

5. What are the major causes of infant mortality in Portugal?

6. Do you think the maternity unit closures had any impacts on inequalities in access to care for pregnant women and mothers, geographic or otherwise?

7. Do you think the maternity unit closures had any impacts on infant mortality?

8. Would you change anything about the policy that was implemented in 2006, and why?

9. Since the maternity unit closures, has it become easier or more difficult for pregnant women to choose and access the maternity unit that they want or need?

10. What impact did the 2006 closures have on the survival of particularly vulnerable babies, such as pre-term, very pre-term and very low birth weight babies?

11. Please add any further comments that you'd like to make:

<u>Supplementary Material 5 (S5)</u>

Survey Sent to Mothers

1. What are the strengths of the maternal and perinatal healthcare system in Portugal today, and what improvements could be made?

2. When did you give birth? At the time(s) of giving birth, what region of Portugal did you live in? Did you live in an urban or rural area?

3. How close did you live to your nearest maternity unit? Were you closer to a unit that was closed in 2006 than you were to the unit where you delivered your baby?

4. Where did your pregnancy-related consultations happen (for example, in your primary healthcare centre, with a private doctor, in a hospital with a maternity unit that was later closed, in the hospital where you delivered your baby)? How regular were the consultations?

5. Where did you have scans during your pregnancy? How many did you have and how many weeks pregnant were you for each one (if you can remember)?

6. Did you consider moving or choose to move house in order to be closer to a maternity unit before giving birth?

7. If you don't mind me asking, how were your pregnancy (or pregnancies) and birth(s)? Were there any complications at any time?

8. Did you feel that you were given sufficient information about giving birth in the hospital, and did you feel that you had freedom of choice of hospital? If not, why not or what could have been done to improve this?

9. Were you satisfied with the care you received when giving birth and the access you had to that care? If not, why not or what would you have changed about the situation?

10. Would you change anything about the policy which was implemented at the time and the maternity unit closures that happened as a result?

11. Please add any further comments that you'd like to make:

Supplementary Material 6 (S6)

Participant Information and Informed Consent Form

Study Participant Information and Informed Consent Form

Informed Consent Form for: policymakers at the time of the 2006 commission on maternal and neonatal health in Portugal, researchers in the areas of infant mortality, maternal and perinatal healthcare and healthcare policy in Portugal, healthcare workers at the time of the 2006 commission and mothers who have given birth in Portugal since the 2006 commission was implemented, who we are inviting to participate in the study "Avoidable Infant Mortality in Portugal: A Scoping Review and Policy Analysis"

Name of Principle Investigator: Morgan Weiland

Name of Organisation: Maastricht University and Coimbra University

Name of Project: Avoidable Infant Mortality in Portugal: A Scoping Review and Policy Analysis

This Informed Consent Form has two parts:

- **Information Sheet (to share information about the study with you)**
- **Consent Form (for signatures if you choose to participate)**

You will be given a copy of the full Informed Consent Form.

Part I: Information Sheet

Introduction

My name is Morgan Weiland, and I a student of the Global Health MSc program at Maastricht University. For my thesis I am undertaking research in healthcare policy and infant mortality in Portugal, with the University of Coimbra. You do not have to decide today whether or not you will participate in the research. Before you decide, you can talk to anyone you feel comfortable with about the research. This consent form may contain words that you do not understand. Please ask me to stop as we go through the information and I will take time to explain. If you have questions, later you can ask them of me then. I will ask you to sign the consent form before beginning the interview.

Purpose of the research

Infant mortality refers to the death of children below the age of 1 year. Avoidable infant mortality refers to infant deaths that, given current medical knowledge and technology, could have been avoided by the healthcare system through prevention and treatment. We want to find ways to stop avoidable deaths from happening. We believe that you can help us by sharing your knowledge and experiences of maternal care in Portugal. We want to know how healthcare policies have impacted access to maternal and perinatal care.

Type of Research

This research will involve your participation in an online interview either over email or using Maastricht University's account on Zoom.

Participant Selection

You are being invited to take part in this research because we feel that your knowledge and experience of healthcare can contribute greatly to our understanding of the wider impacts of healthcare policies.

Voluntary Participation

Your participation in this research is entirely voluntary. It is your choice whether to participate or not. You can change your mind about participating at any time. If you change your mind during the interview or after the interview before analysis has been conducted, your data will be destroyed and you will not be included in the analysis. If the analysis has already been conducted then it will not be possible to remove you from the analysis but your data will be destroyed.

Procedures

We are asking you to help us learn more about healthcare for pregnant and labouring women in Portugal and the relevant policies. We are inviting you to take part in this research project as we believe your experience or knowledge of this could help us understand this part of the healthcare system better. If you accept, you will be asked to participate in an interview conducted online either over email or via Maastricht University's account on Zoom.

Interviews will take place with myself. If you do not wish to answer any of the questions during the interview, you may say so and the interviewer will move on to the next question. No one else but the interviewer will be present unless you would like someone else to be there. The information recorded, whether as an audio recording of the interview or in the form of notes made by myself during the interview, is confidential, and no one else except my supervisor, Eva Pilot, will access to the information documented during your interview. You will be asked whether you give consent for the interview to be audio recorded. If you give consent, the entire interview will be tape-recorded, but no-one will be identified by name on the tape. This will be using a physical tape, which will be kept in a locked drawer at the home of the interviewer. The information recorded is confidential, and no one else except Eva Pilot will have access to the tapes. The tapes will be destroyed after 6 weeks, after they have been transcribed into Microsoft Word documents. These documents will only be accessible by the researcher, and will be destroyed after 5 years.

Duration

The interview will take around 30 minutes.

Risks

There is a risk that you may share some personal or confidential information by chance, or that you may feel uncomfortable talking about some of the topics. However, we do not wish for this to happen. You do not have to answer any question or take part in the interview if you feel the question(s) are too personal or if talking about them makes you uncomfortable.

Benefits

There will be no direct benefit to you, but your participation is likely to help us find out more about the role of healthcare policy in access to healthcare for pregnant and labouring mothers.

Reimbursements

You will not be provided any incentive to take part in the research.

Confidentiality

Your confidentiality will be maintained at all times. Files containing raw data which could be used to identify you will be kept in an encrypted, password-protected folder. This data will be anonymised before being analysed, and will only be kept outside of the encrypted folder in the

anonymised form. You will be known anonymously by your role and a number assigned to you by a random number generator, for example: policymaker 2; researcher 1; mother 3. Data collected from your participation will be anonymised so that you cannot be identified from it. Files containing data will be encrypted.

Sharing the Results

Your anonymity will be ensured throughout the research. Only the principle investigator and, upon request, the research team, will have access to interview recordings or notes. Each participant will receive a summary of the results. The findings of this research may be shared more widely, for example through publications and conferences.

Right to Refuse or Withdraw

You do not have to take part in this research if you do not wish to do so, and choosing to participate will not affect your job or job-related evaluations in any way, as all data that is public or widely available will be anonymised and it will not be possible for you to be identified. You may stop participating in the interview at any time that you wish. If you withdraw during the interview or after the interview before analysis has been conducted, your data will be destroyed and you will not be included in the analysis. If the analysis has already been conducted then it will not be possible to remove you from the analysis but your data will be destroyed.

Who to Contact

If you have any questions, you can ask them now or later. If you wish to ask questions later, you may contact either of the following:

Morgan Weiland – email: morganweiland@btinternet.com

Eva Pilot – email: eva.pilot@maastrichtuniversity.nl

This proposal has been reviewed and approved by the FHML-REC (Ethics Review Committee Health, Medicine and Life Sciences) of Maastricht University, which is a committee whose task it is to make sure that research participants are protected from harm. This is a special provision for Global Health Research within FHML. If you wish to find about more about the FHML-REC, contact the Research Office of the Faculty of Health, Medicine and Life Sciences at Maastricht University:

Maastricht University

Research Office FHML

P.O. Box 616

6200 MD, Maastricht, NL

+31 43 38 71 350

info-researchoffice@maastrichtuniversity.nl

Part II: Certificate of Consent

Statement by the participant

I have been invited to participate in research about healthcare and avoidable infant mortality in Portugal. I consent to my interview responses being used in this study. I have read the foregoing information, or it has been read to me. I have had the opportunity to ask questions about it and any questions I have been asked have been answered to my satisfaction. I consent voluntarily to be a participant in this study.

Print Name of Participant__________________

Signature of Participant ___________________

Date ___________________________

Day/month/year

If illiterate

I have witnessed the accurate reading of the consent form to the potential participant, and the individual has had the opportunity to ask questions. I confirm that the individual has given consent freely.

Print name of witness____________

Signature of witness _____________

Date ________________________

Day/month/year

Recording

I consent to my interview being recorded.

Print Name of Participant__________________

Signature of Participant ___________________

Date ___________________________

Day/month/year

If illiterate

I have witnessed the accurate reading of the consent form to the potential participant, and the individual has had the opportunity to ask questions. I confirm that the individual has given consent freely for their interview to be recorded.

Print name of witness____________

Signature of witness _____________

Date ________________________

Day/month/year

Statement by the researcher/person taking consent

I have accurately read out the information sheet to the potential participant, and to the best of my ability made sure that the participant understands that the following will be done:

1. The participant will take part in an interview or survey and their responses will be recorded and used in this study.
2. The results of the study will be communicated to the participant before the study is submitted as a thesis or made public in a published work.

I confirm that the participant was given an opportunity to ask questions about the study, and all the questions asked by the participant have been answered correctly and to the best of my ability. I confirm that the individual has not been coerced into giving consent, and the consent has been given freely and voluntarily.

A copy of this ICF has been provided to the participant.

Print Name of Researcher/person taking the consent________________________

Signature of Researcher /person taking the consent__________________________

Date ___________________________

Day/month/year
